# Reliability and oncological safety of pedicled skin island therapeutic mammoplasty as an alternative to Grisotti mastopexy for centrally located breast cancer patients

**DOI:** 10.1186/s12957-026-04399-z

**Published:** 2026-05-14

**Authors:** Nagm Eldin Abu Elnga, Mohamed K Safina, Mahmoud Refaat Shehata, Mahmoud T. Ayoub, Ahmed Soliman

**Affiliations:** 1 General Surgery Department, Faculty of medicine, Assiut University, Assiut, Egypt; 2 General and oncology surgery Department, Faculty of medicine, New Valley University, Assiut, 71526 Egypt

**Keywords:** Pedicled skin island therapeutic mammoplasty, Centrally located breast cancer, Nipple-areolar complex, Oncoplastic breast surgery, Grisotti flap, Breast conservation

## Abstract

**Background:**

Centrally located breast cancers (CLBC) involving the nipple-areolar complex (NAC) pose a significant challenge for breast-conserving surgery, often necessitating mastectomy. The Grisotti flap, while a valuable oncoplastic technique, has limitations in vascular reliability and applicability, especially post-neoadjuvant chemotherapy. We introduce a novel “Pedicled Skin Island Therapeutic Mammoplasty” (PSI-TM) as an alternative.

**Methods:**

This single-center retrospective study analyzed 23 consecutive patients with CLBC infiltrating or fixed to the NAC who underwent PSI-TM between April 2018 and June 2023. All patients had medium-to-large breast volumes (Cup C/D). Data on demographics, tumor characteristics, surgical outcomes, complications, aesthetic results, and oncological safety were collected and analyzed.

**Results:**

The cohort was predominantly premenopausal (82.6%) with invasive ductal carcinoma (87.0%). Most patients (73.9%) were treated post-neoadjuvant chemotherapy. The overall complication rate was 21.7% (5/23), all Clavien-Dindo Grade I-II, with no returns to the operating room. The combined ratings from the surgeon and independent observer were ‘Good’ or ‘Very Good’ in 73.9% of cases (17/23). Over a median follow-up of 50 months, there were no instances of positive margins, local recurrence, or distant metastasis.

**Conclusion:**

PSI-TM is a feasible and safe oncoplastic technique for CLBC with NAC involvement. It may offer improved vascular security compared to the traditional Grisotti flap based on theoretical anatomical advantages, leading to low complication rates, excellent aesthetic results, and encouraging early oncological outcomes, even in a post-neoadjuvant setting.

## Introduction

Centrally located breast cancers, particularly those involving or in close proximity to the nipple-areolar complex (NAC), constitute about 5% to 20% of all breast cancer cases. However, successfully removing these tumors with adequate margins while maintaining an acceptable breast appearance using conventional breast-conserving surgery approaches has proven difficult. In past years, mastectomy was frequently recommended as the standard treatment for centrally located breast cancers to ensure complete excision and mitigate the high risk of positive margins and local recurrence associated with conventional lumpectomy in this anatomically complex region [1, 2].

Grisotti flap mammoplasty, which was first introduced in 1984, is considered a pivotal advancement in onco-plastic breast surgery [3]. Grisotti flap mammoplasty is defined as a rotational advancement flap technique rotated from the lower inner quadrant to close the defect caused by excising a tumor centrally with the NAC. It provides, however, the considerable advantage of breast conservation, combined with good cosmetic results, in comparison with mastectomy for central tumors. It has, however, recognized limitations, such as limitations of vascular supply of the flap, particularly if its pedicle is narrow or if a large flap is necessary, leading to flap or fat tissue necrosis, dehiscence, and poor cosmetic results [4, 5].

The need for a medial pedicle rotation-advancement breast flap is also recognized as being technically demanding due to breast size, width of base, or ptosis, as well as being ineffective for managing tumors that may have attachment to the NAC located higher than the tumor. Potential for oncological safety issues exists due to proximity of pedicle to tumor site and also regarding assessment of margins through the complexity of the flap [6, 7].

Over the years, contemporary approaches for dealing with breast cancers, such as volume displacement techniques (round block, batwing, etc.) and volume replacement techniques (LICAP, TDAP, etc.), have extended the possibilities for dealing with central tumors [8, 9]. However, breast cancers that invade NAC parenchymal tissue itself or are fixed superiorly to NAC can still be challenging for BCS. NSM is also a good solution in such cases, but there is a risk of nipple necrosis, and it is a more extensive procedure than BCS or reconstructive surgery [10].

In order to overcome these drawbacks, we propose a novel technique known as “Pedicled Skin Island Therapeutic Mammoplasty” (PSI-TM). This novel technique conceptualizes the idea of tumor resection in a different manner. When tumors are attached to or infiltrate the NAC, PSI-TM involves a pre-designated inferiorly based “dermo-glandular pedicle” with a “skin island”. This technique of tumor resection differs significantly from that of Grisotti’s method in that it involves a “robust inferiorly based pedicled flap with a skin island” [11, 12].

The integrated skin island can be tailor-designed to reconstruct the exact NAC position and contour without subsequent tattooing or grafting in most patients, further enhancing aesthetic symmetry.

Breast cancer exhibits significant molecular heterogeneity, with distinct subtypes influencing tumor biology, response to neoadjuvant therapy, and surgical outcomes [13]. Understanding these biological markers is essential when planning oncoplastic procedures, particularly in the post-neoadjuvant setting where tumor regression patterns may affect margin status and flap viability.

The current report describes the early experience with PSI-TM in a consecutive series of 23 patients. The goal is to provide evidence concerning technical feasibility, short-term oncological safety, as well as early functional and aesthetic efficacy for this novel technique, in order to establish it as a viable, if not superior, alternative to the Grisotti flap for this particularly challenging subset of breast cancer patients.

## Patients and methods

### Study design and ethical approval

This study is a single-center retrospective analysis of prospectively collected data in the first 23 consecutive patients with centrally located breast cancer (CLBC) infiltrating or fixed to the NAC and treated with PSI-TM between April 2018 and June 2023 in the General surgery department at Assiut university. The research was approved by the Ethics Committee of Assiut university (IRB: 04-2025-300615), and written informed consent was obtained from all patients for both the surgical procedure and the use of clinical photographs for research and publication purposes. The prospective data collection was conducted through a dedicated surgical database with specific consent for future research use. This Level IV evidence study is designed to demonstrate technical feasibility and early outcomes of PSI-TM, and to generate hypotheses for future comparative research. 

### Patient selection & surgical indication

This study consisted of a consecutive series of 23 patients with centrally located breast tumors—histologically confirmed invasive carcinoma or ductal carcinoma in situ (DCIS) within 1 cm of the nipple-areola complex (NAC) or with NAC infiltration—who were treated with pedicled skin island TM. The interventions were all conducted by the same investigator.

The technique was offered as a breast-conserving therapeutic option, either initially or following neoadjuvant chemotherapy (NACT), to patients meeting the following inclusion criteria: patients with medium-to-large breast volume (bra cup size C or greater) with moderate-to-severe ptosis (Regnault grades II-III), rendering the patients suitable for mammoplasty-based reconstruction approaches; patients for whom mastectomy would otherwise have been indicated following multi-disciplinary tumor board consensus for achieving breast-conserving therapy instead of mastectomy; for whom full clinical and pathological records are accessible for retrospective analysis; and for whom a follow-up period of 24 months or greater post-surgery is accessible in order to investigate short-term oncological safety, surgical complications, and aesthetic result potential.

Exclusion criteria included: small breast volume (Cup A-B), minimal or no ptosis (Regnault grade 0-I), inflammatory breast cancer, extensive skin involvement, prior breast surgery that would compromise pedicle viability, and medical comorbidities precluding oncoplastic surgery.

### Surgical technique

**Fig. 1:**
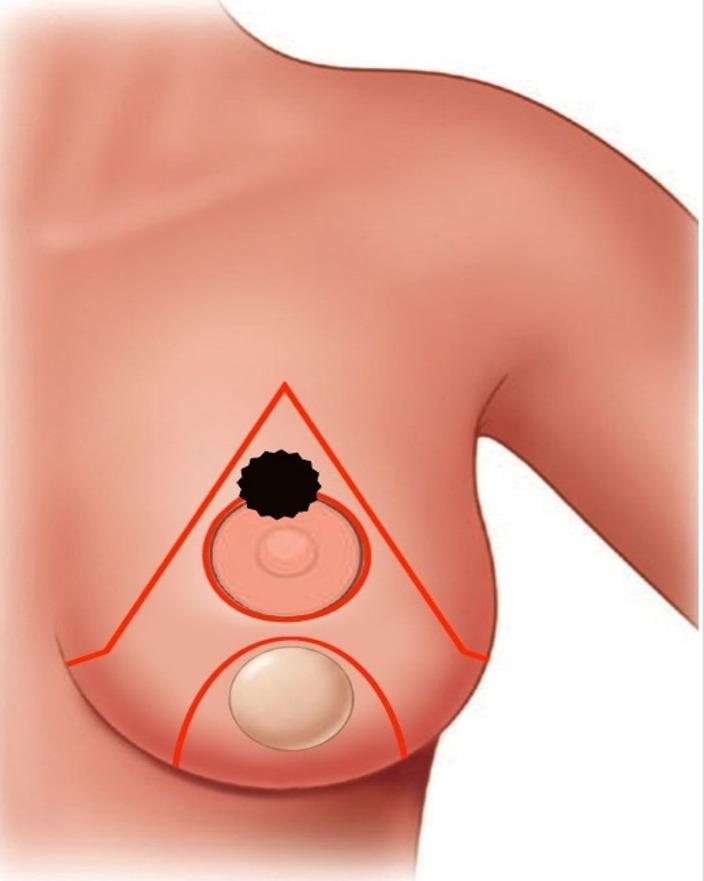
Preoperative surgical marking of the breast demonstrating the inferior pedicle flap design (red lines) with precise delineation of the new inframammary fold (IMF) and a 4–5 cm circular skin island (inset) designated for neo-areolar reconstruction. The magnified inset illustrates the tumor (black) within the excised tissue, emphasizing its anatomical relationship to surrounding structures and guiding margin assessment in alignment with Wise-pattern reduction mammoplasty principles

**Fig. 2:**
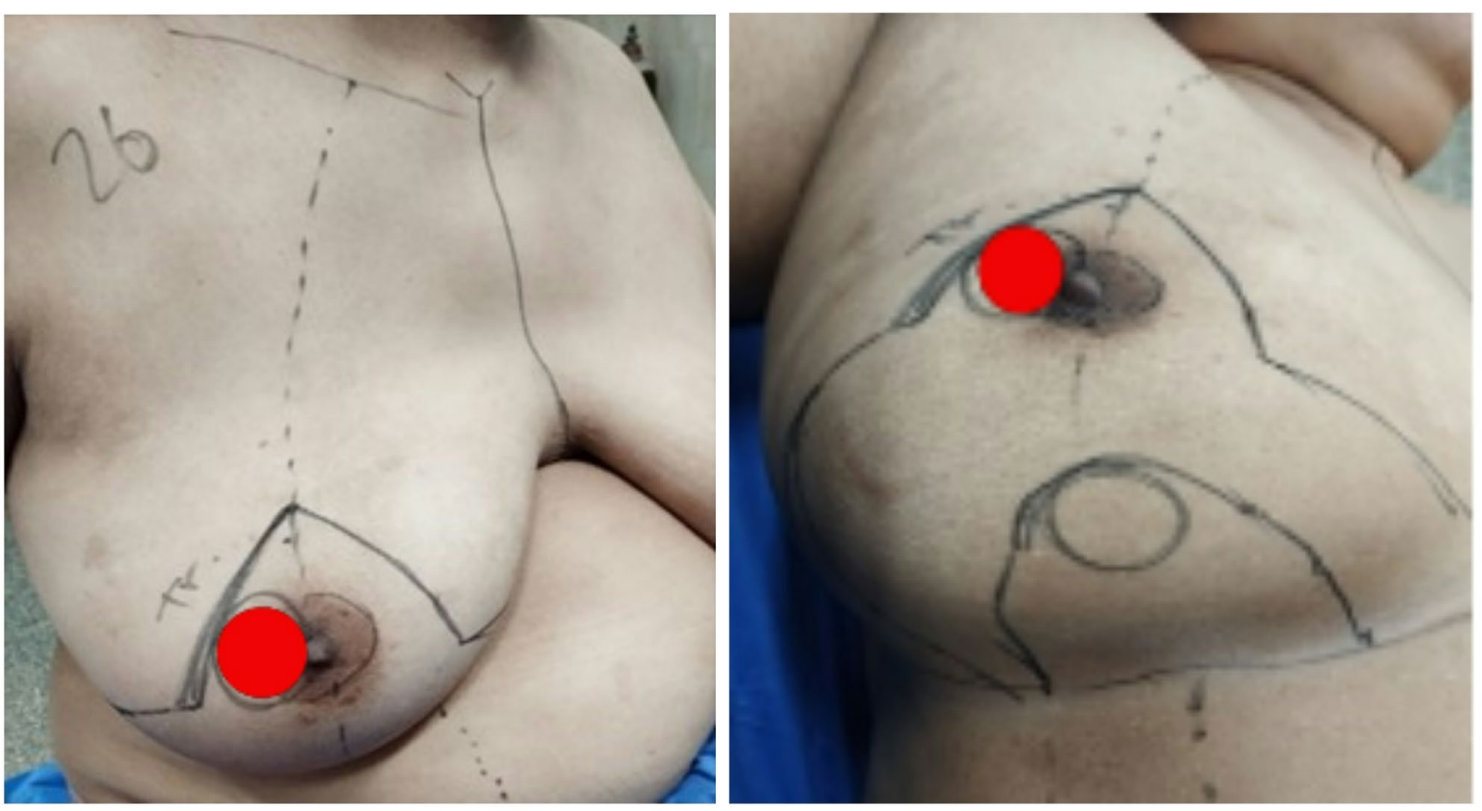
A photo showing the initial marking of the sternal notch, midlines, inframammary folds (IMF), and the breast meridian. The anticipated new position for the neo-NAC (skin island) is marked at the apex of the breast mound. The central tumor infiltrating the nipple-areola complex (NAC) is marked by a red oval for planned en bloc resection

**Fig. 3:**
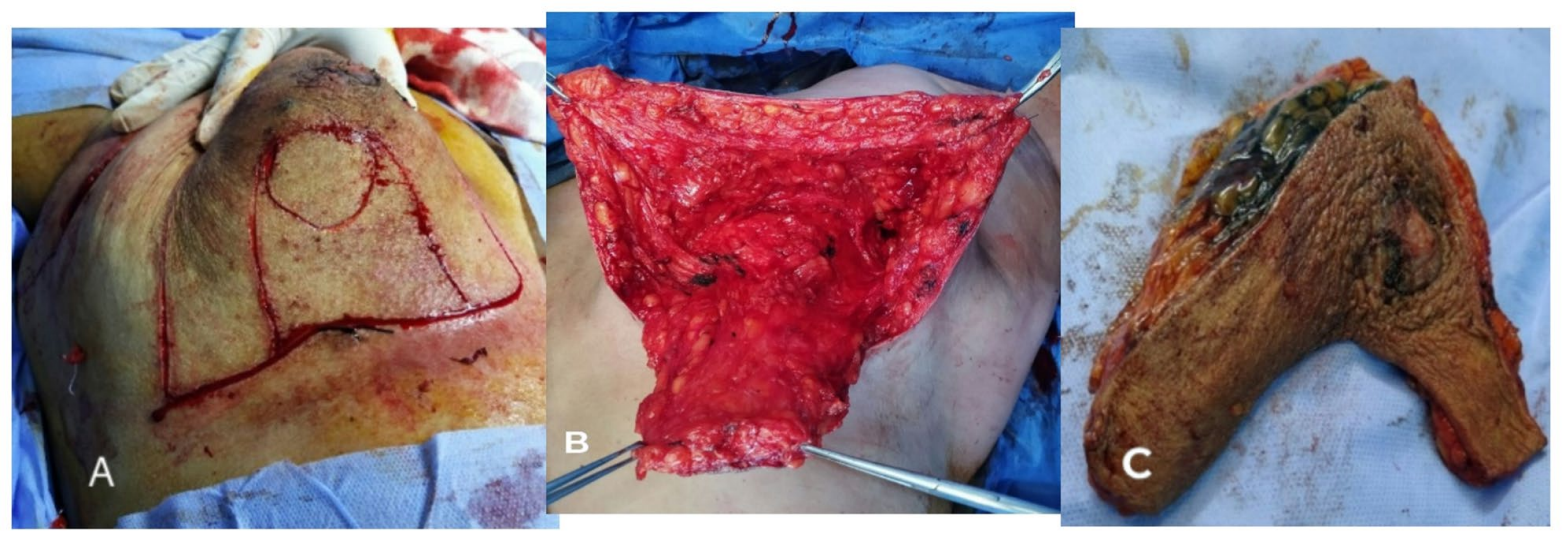
Intraoperative photographs demonstrating key steps of pedicled skin island therapeutic mammoplasty. **A** Preoperative surgical markings on the breast showing the inverted-T (Wise-pattern) incision and central tumor/NAC area planned for en bloc resection. **B** the tumor bed after en bloc resection of centrally located breast cancer with nipple-areolar complex, prior to PSI-TM reconstruction. **C** The resected central breast specimen containing the nipple-areola complex (NAC), the tumor, and a cylinder of surrounding parenchyma

**Fig. 4:**
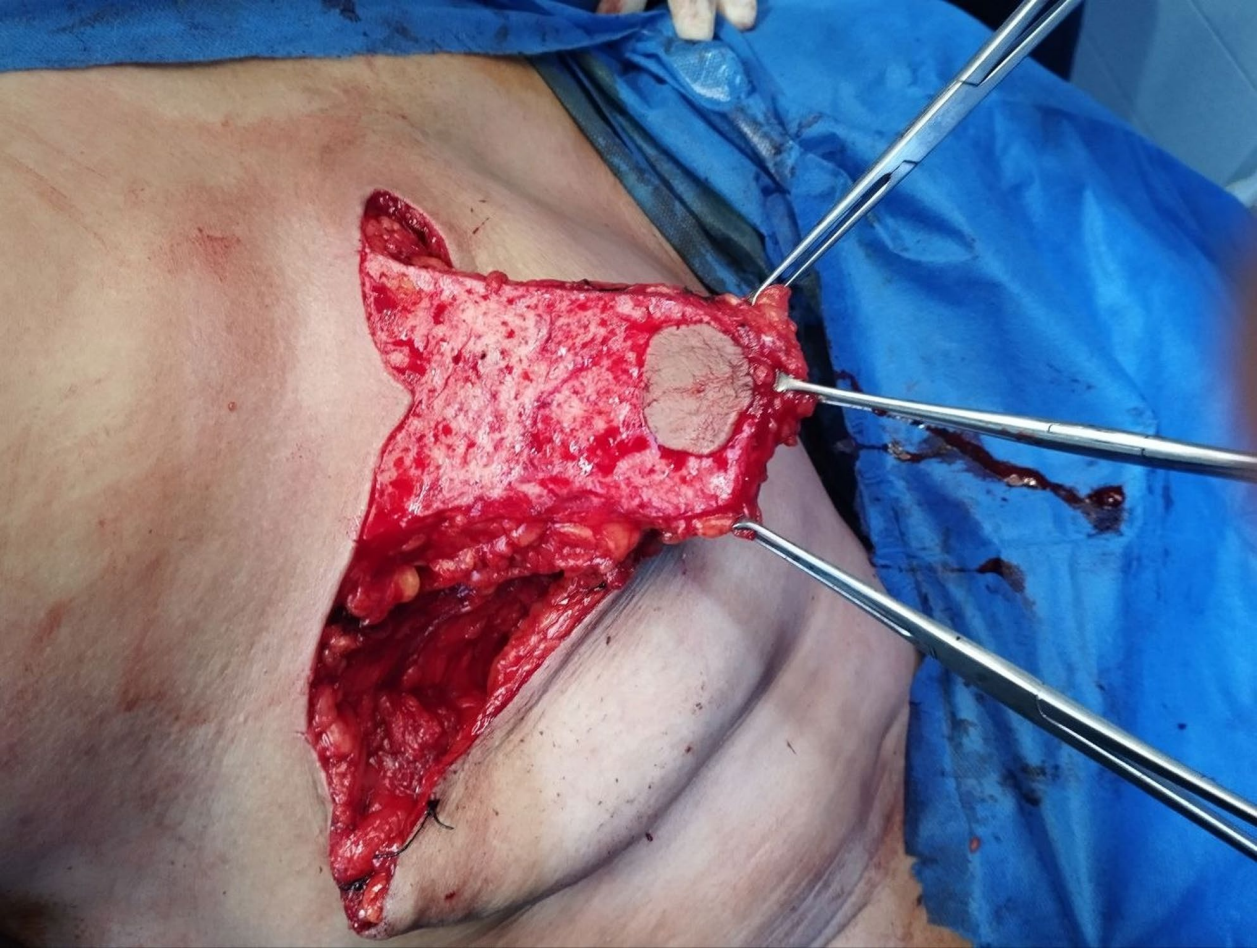
A photo showing the area of the inferior pedicle, *except for the central skin island*, is being de-epithelialized. The inferiorly based dermo-glandular pedicle being elevated off the pectoralis fascia. The robust pedicle with the intact, vascularized skin island at its superior end should be clearly visible

**Fig. 5:**
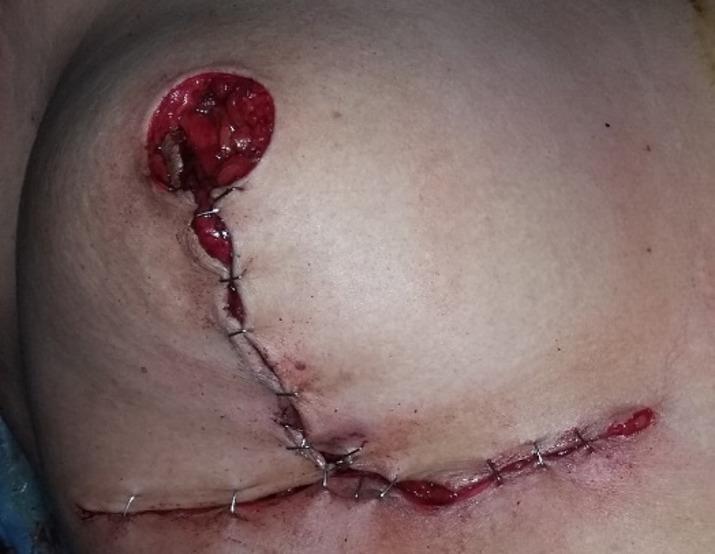
A photo showing the approximation of the breast pillars (medial and lateral flaps) and the typical Wise-pattern skin closure, creating the new breast cone

**Fig. 6:**
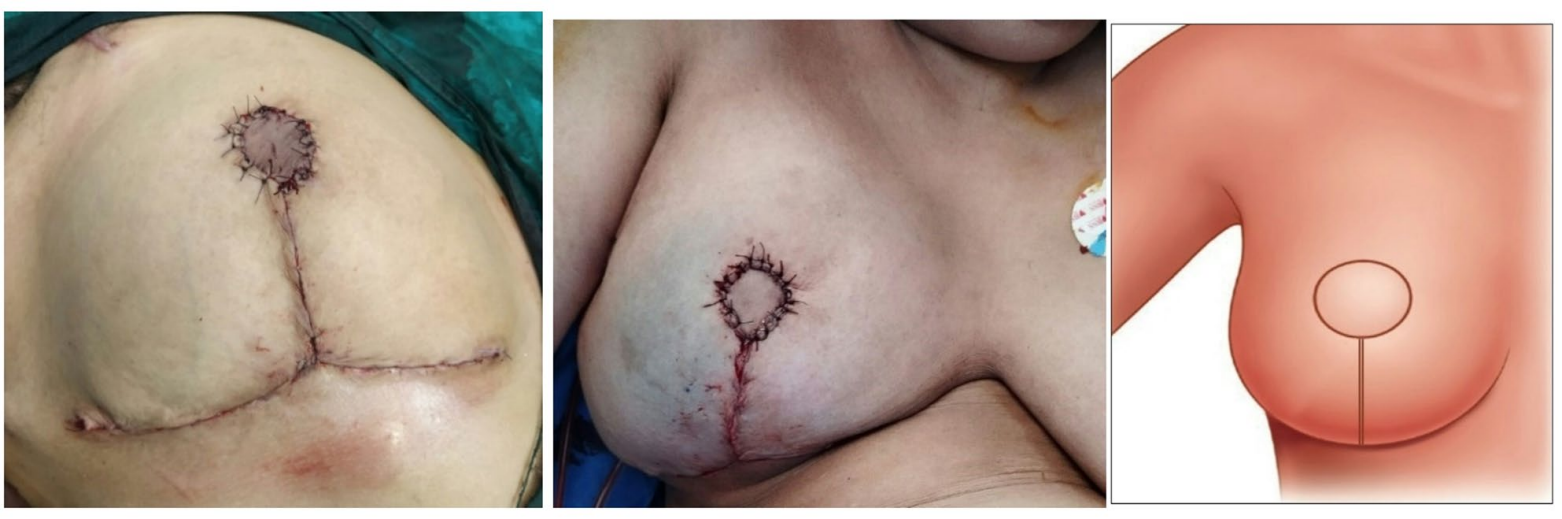
The inferior dermo-glandular pedicle has been rotated superiorly into the central defect. The cutaneous island, which will form the new areolar platform, is secured at the apex of the reconstructed breast mound. The wound is closed in a standard Wise-pattern (inverted-T) fashion. The vertical and inframammary fold incisions are approximated, demonstrating the immediate reduction mammoplasty shape achieved on the operative side

**Fig. 7:**
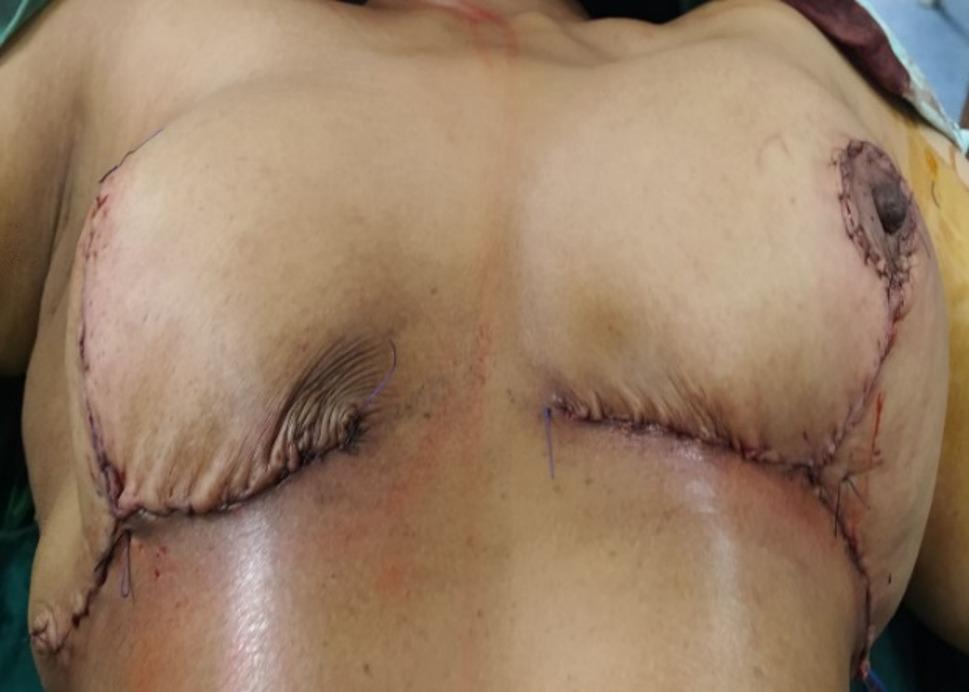
A standard Wise-pattern, inferior pedicle reduction mammoplasty is performed on the opposite breast to achieve symmetry in volume, shape, and nipple position

- Indications and contraindications are detailed in the inclusion and exclusion criteria.
- Step 1 – Preoperative marking: With the patient upright, the breast meridian, inframammary fold (IMF), and the Wise-pattern keyhole are marked. The inferior pedicle is outlined with a base width of 8-10 cm, extending from the IMF to the planned neo-NAC position (fig. 1) . The superior border of the pedicle incorporates a circular or oval skin island (4-5 cm diameter) that will become the neo-areola. The location of the nipple-areola complex (NAC) is marked along the meridian of the breast. The pedicle's axis is oriented vertically along the breast meridian to capture perforators from the internal mammary artery (medially) and intercostal vessels (laterally) (fig. 2).
- Step 2 – Incision and resection: The marked incisions are made with a scalpel. NAC, tumor mass, and surrounding tissues are excised as a single mass in a central cylinder fashion. The tissue is then sent for pathological evaluation (fig. 3).
- Step 4 – Pedicle elevation: The inferior dermo-glandular pedicle is dissected off the pectoralis fascia from inferior to superior, preserving the perforating vessels (predominantly from the internal mammary artery medially and intercostal vessels laterally). Care is taken to maintain a pedicle thickness of at least 2-3 cm to ensure adequate perfusion.
- Step 5 – De-epithelialization: The inferior pedicle is deepithelialized except its central circular portion, which constitutes the new areolar bed - for the purpose of later tattooing or nipple reconstruction (fig. 4).
- Step 6 – Pillar closure and remodeling: The medial and lateral breast pillars are approximated with 2-0 absorbable sutures to reconstruct the breast cone. The Wise-pattern skin flaps are closed in layers over a closed suction drain (fig 5).
- Step 7 – Flap rotation and inset: The pedicle is rotated superiorly into the central defect. The skin island is positioned at the breast apex corresponding to the original NAC location. The pedicle is secured with interrupted absorbable sutures to the surrounding parenchyma (figs. 6 and 7). Drains placed bilaterally, multilayer closure with absorbable sutures.
- Critical Technical Points: - Pedicle base must remain sufficiently wide (minimum 8 cm) to ensure vascular supply - Central skin island must be carefully preserved during de-epithelialization - All dissections must remain superficial to pectoralis fascia to preserve perforators - Avoid tension on pedicle during rotation and inset.
- All procedures were performed by a single senior surgeon experienced in oncoplastic techniques. PSI-TM requires familiarity with Wise-pattern mammoplasty and perforator-sparing dissection; adoption by less experienced surgeons should follow structured training.

### Data collection

Data were also collected electronically from medical records and a study database designed to collect data prospectively for surgeries. Variables collected included patient demographics, tumor characteristics (size, histology, grade, and receptor status), and operative details (operative duration, and estimated blood loss). Histopathological outcomes, specifically margin status and lymph node status, were recorded. Data on all forms of adjuvant treatment, including chemotherapy, radiation, and immunotherapy, was also collected. Complications, length of hospital stay, readmissions, and reoperations, according to the Clavien-Dindo system, were also noted.

Aesthetic outcomes have been assessed using a non-validated multi-rater system of evaluation comprising of the patient, operating surgeon, and independent observer. Physical examination and a comparative study of pre- and post-surgery photographs of patients were carried out by the surgeon and independent observer.

Assessment of three major parameters—breast shape, nipple-areola complex positioning, and symmetry—has been performed on a 4-point Likert scale. Higher scores indicate better results, with 4 indicating very good (perfect shape/form; perfect appearance consistent with a normal appearance of the parameters under evaluation), 3 representing very good results/near perfect shape/forms, 2 indicating good with imperfect forms/very good appearance within acceptable limits, and 1 indicating very poor results/asymmetry/as undesirable appearance. For each of these parameters, a final value was obtained by averaging out the surgeon’s and independent examiner’s results.

Patient satisfaction was assessed via a simple informal questionnaire administered at the 12-month follow-up visit (very satisfied / satisfied / disappointed / regretted the decision).

While this custom scale provided a practical framework for initial assessment, we acknowledge that it lacks formal validation. Future studies should incorporate validated instruments such as the BREAST-Q or Harvard/Harris scale to strengthen aesthetic outcome assessment.

### Statistical analysis

For the analysis of the results, continuous variables are presented as median and range, and categorical variables as frequencies and percentages. Given the small sample size (*N* = 23) and zero events for the primary oncological outcomes (positive margins, local recurrence, distant metastasis), data are presented descriptively. No formal hypothesis testing or survival analysis was performed.

### Follow up protocol

**Fig. 8:**
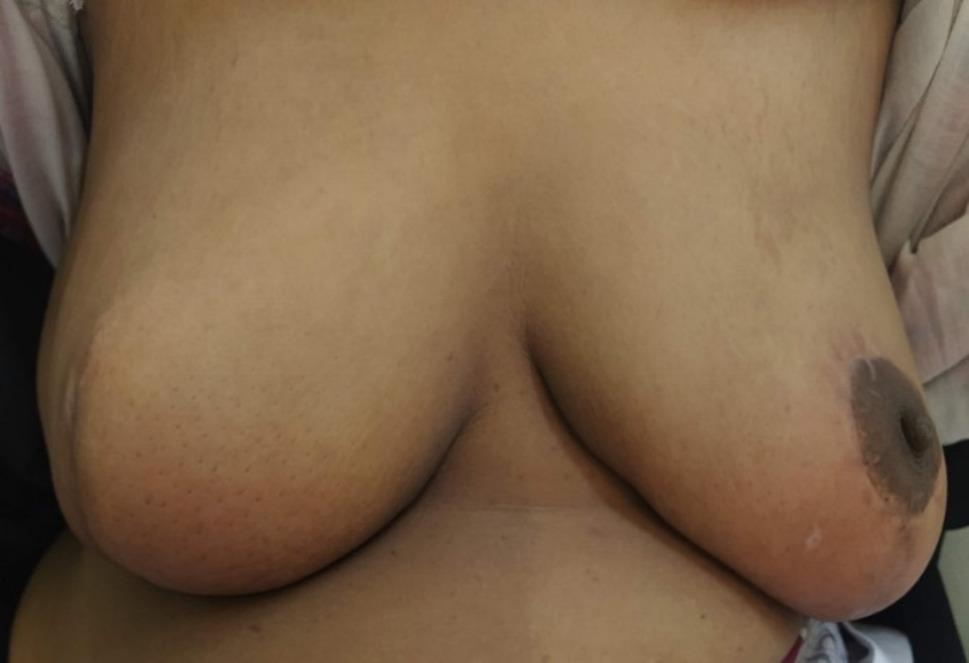
A photo from a follow-up visit (e.g., 6–12 months post-op) showing the fully healed breast. and the overall cosmetic result are displayed

Patients were followed according to the standard institutional breast cancer protocol. This means that the patients were checked clinically every 3 to 6 months for 2 years, then every 6 to 12 months afterwards, along with an annual mammography. Other imaging procedures were done per standard clinical guidelines. The complications were also monitored by recording observations during scheduled follow-up sessions at 2 weeks, 3 months, 6 months, 1 year, and then yearly. Patient-reported outcomes were collected at baseline (preoperatively), and at 3 months and 1 year postoperatively when feasible (Fig. 8).

## Results

This cohort consisted of 23 relatively young and premenopausal patients with a median age of 44 years, which correlates with cohorts of patients suitable for oncoplastic breast-conserving surgeries The majority had invasive ductal carcinoma (87.0%) staged predominantly as T1/T2 (87%) with stages I and II (86.9%) in accordance with American Joint Committee on Cancer Staging definitions. Interestingly, most patients (73.9%) underwent surgeries following neoadjuvant chemotherapy, affirming the applicability of this procedure in a downsizing setting. All these patients had medium-to-large breast volumes categorized into Cup C or D; this is a necessary criterion as a prerequisite for the described mammoplasty-based approach procedures (Table 1).

**Table 1:** Demographic and clinicopathological characteristics of the cohort (*N* = 23)

Variable	*N* (%) or Median [Range]
Age (years)	44 [27–55]
Menopausal Status	
Premenopausal	19 (82.6%)
Postmenopausal	4 (17.4%)
Pathology	
Invasive Ductal Carcinoma	20 (87.0%)
Invasive Lobular Carcinoma	2 (8.7%)
Paget’s Disease	1 (4.3%)
Clinical T Stage	
T1	12 (52.2%)
T2	8 (34.8%)
T3	3 (13.0%)
Tumor Stage	
Stage I	11 (47.8%)
Stage II	9 (39.1%)
Stage III	3 (13.0%)
Surgical Context	
Upfront Surgery	6 (26.1%)
Post-Neoadjuvant Chemotherapy	17 (73.9%)
Axillary Surgery	
Sentinel Lymph Node Biopsy	18 (78.3%)
Axillary Lymph Node Dissection	5 (21.7%)
Breast Cup Size	
Cup C	13 (56.5%)
Cup D	10 (43.5%)
Adjuvant treatment	
Radiotherapy	23 (100%)
Hormonal Therapy	15 (65.2%)
(Post-NACT) Chemotherapy	6 (26.1%)
Targeted Therapy	7 (30.4%)

Post-operative complications included five patients (21.7%), and in some cases, patients experienced more than one post-operative complication. The most prominent wound complications arose from minor dehiscences observed in four patients, corresponding to 17.4% of the total number of cases, which were mostly associated with the T-junction area of the incision site as described for the Wise pattern incision.

Three patients developed postoperative seromas in the dependent portion of the breast. All were asymptomatic and detected on routine clinical examination. Management involved observation for spontaneous resolution or, in one persistent case, a single ultrasound-guided aspiration, after which complete resolution was obtained. Three patients were diagnosed with fat necrosis, all of them were expectantly managed with reassurance and clinical monitoring.

One patient (4.3%) had partial skin necrosis of the distal edge of the skin island flap. This manifested as a small, localized eschar about 1 cm in diameter. The necrotic tissue was left to fully demarcate and was treated conservatively with regular dressings; this ultimately led to complete wound healing without the need for surgical debridement or compromise of the viability of the underlying flap.

All complications were classified as Clavien-Dindo Grade I or II, implying that all were successfully treated using pharmacological interventions or minor bedside procedures. Crucially, however, none of the complications led to total flap loss, required reoperation, or resulted in any delay in the onset of adjuvant radiotherapy and systemic therapies, supporting the technique’s acceptable safety profile (Table 2).

**Table 2:** Postoperative complications (*N* = 23)

Complication	Number of Events	Patients Affected, *n* (%)
Any Complication	-	5 (21.7%)
Wound Dehiscence	4	4 (17.4%)
Breast Seroma	3	3 (13.0%)
Fat Necrosis	3	3 (13.0%)
Skin Necrosis	1	1 (4.3%)

The cumulative result of the surgeon’s and observer’s ratings was ‘Good’ or ‘Very Good’ in 73.9% (17/23), with only a single case (4.3%) rated as ‘Poor’. Patient-reported satisfaction with aesthetic results was highly positive, with all patients (100%) scoring themselves as ‘Satisfied’ or ‘Very Satisfied’ with the results.” The degree of congruence between expert and patient assessment serves to reflect upon the technique’s consistency with regard to meeting higher aesthetic expectations, alongside its oncological goals (Table 3).

**Table 3:** Aesthetic and patient-reported outcomes

Assessment	Category	*n*	%
Professional Assessment	Very Good / Good	17	73.9%
	Satisfactory	5	21.7%
	Poor	1	4.3%
Patient Assessment	Very Satisfied / Satisfied	23	100%
	Disappointed / Regretted	0	0%

In a median follow-up period of 50 months (mean, 59.2 months; range, 24 to 84 months), there was no incidence of reoperation, positive margins, local recurrence, or distant metastasis among the 23 patients, indicating encouraging short-term oncological results. Obviously, the intraoperative frozen section may be a distinctive advantage because all patients had negative margins, which may account for the satisfactory results.

Likewise, adjuvant therapies were given in accordance with multiple disciplines, and all 23 cases were given radiotherapy, representing 100.0% of cases. Hormonal therapy was noted to be the most common form of systemic therapies, at 65.2%, targeted therapy and chemotherapy were given in 30.4% and 26.1% of cases, respectively. This comprehensive adjuvant approach, integrated with the oncoplastic technique, supports optimal disease control, as evidenced by the absence of recurrences in the study period, though comparisons have to be made to establish benefits over traditional techniques.

## Discussion

The present retrospective case series evaluates the feasibility, early oncological outcomes, and efficacy of the pedicled skin island therapeutic mammoplasty (PSI-TM) in 23 patients with centrally located breast cancer (CLBC) or nipple-areola complex (NAC)-infiltrating tumors.

Unlike the Grisotti flap—which relies on a rotational advancement of glandular tissue from the lower inner quadrant with a largely random-pattern blood supply—PSI-TM employs a formal inferiorly based dermo-glandular pedicle with an integrated skin island. This design provides a more robust axial vascular supply (via internal mammary and intercostal perforators), allows greater superior reach for tumors attached to or infiltrating the NAC from above, and enables immediate neo-areolar reconstruction without secondary grafting or tattooing (Table 4).

**Table 4:** Conceptual and technical comparison between PSI-TM and grisotti flap mammoplasty^*^

Feature	PSI-TM (present technique)	Grisotti mammoplasty
Pedicle type	Inferiorly based dermo-glandular pedicle	Rotational advancement flap from lower inner quadrant
Skin island	Pre-marked, integrated into pedicle (4–5 cm)	Not an inherent component
Vascular supply	Axial (internal mammary + intercostal perforators)	Random pattern / advancement-dependent
Flap mobility	Superior rotation; excellent reach for high-riding tumors	Limited superior reach
Ideal breast anatomy	Cup C/D, Regnault ptosis II-III	Moderate breast size, variable ptosis
Neo-areolar reconstruction	Immediate via skin island	Often requires secondary tattooing or grafting
Lower pole projection	Preserved (Wise-pattern reduction)	Potential flattening

^*^ This table describes technical differences between approaches, not relative effectiveness. The optimal technique depends on patient anatomy, tumor characteristics, surgeon expertise, and patient preferences. Comparative effectiveness studies are needed to establish relative indications

It is important to emphasize that this study does not provide direct comparative evidence against the Grisotti flap or other oncoplastic techniques. The comparisons drawn with published Grisotti outcomes are indirect and hypothesis generating. A prospective randomized or matched cohort study directly comparing PSI TM with Grisotti mammoplasty would be required to definitively establish comparative effectiveness, safety, and aesthetic outcomes.

The observed postoperative complications were all minor (Clavien Dindo Grade I II) and did not delay adjuvant therapy. When placed in the context of published Grisotti series, where overall complication rates range from 10 20% [14, 15], our findings suggest that PSI TM may offer a favorable safety profile. A 2023 study on extreme oncoplasty with Grisotti procedures reported a 13.9% wound related complication rate [16], while a 2025 randomized trial comparing Grisotti flap with central quadrantectomy noted wound infection in 15% and seroma in 10% of Grisotti patients [17]. The absence of major flap loss or reoperation in our cohort further supports the vascular reliability of the inferiorly based pedicle design.

The theoretical advantage of PSI TM lies in its robust dermo glandular pedicle, which preserves axial blood supply from internal mammary and intercostal perforators. This may be particularly beneficial for superiorly located defects, where traditional advancement flaps (e.g., Grisotti) can be vulnerable to distal ischemia [18]. Moreover, the routine use of intraoperative frozen section contributed to a 0% re excision rate, comparing favorably with reported re excision rates of approximately 4.7% in Grisotti series [19].

Aesthetic results were highly favorable, with most cases rated as good or very good by professional assessors and patient satisfaction being uniformly positive. These outcomes compare well with the Grisotti literature, where cosmetic satisfaction ranges from 85 95% in moderate sized breasts with central defects [20, 21]. However, a 2021 case report on modified Grisotti flaps highlighted limitations in small, non ptotic breasts and noted postoperative asymmetry [18]. PSI TM’s integration of Wise pattern reduction elements allows for more predictable volume redistribution and symmetrization, particularly in patients with ptotic, larger breasts (cup C D). The low proportion of poor results in our series (4.3%) supports this advantage.

Nevertheless, the 100% patient satisfaction rate should be interpreted with caution. It may reflect response bias (e.g., patients’ reluctance to report dissatisfaction to their treating surgeon) or the use of a non-validated questionnaire. Future studies employing validated instruments such as BREAST Q are needed to obtain more robust and comparable aesthetic data.

The oncological results are encouraging but preliminary (Table 5). With a median follow-up of 50 months, no positive margins, local recurrences, or distant metastases were observed. For context, the Grisotti mammoplasty is associated with 5-year local recurrence rates of 2–10% and disease-free survival exceeding 90% in similar cohorts [22, 23]. A 2023 Grisotti study for CLBC reported a short-term recurrence rate of 3.4% [24]. However, the small sample size and absence of events mean that definitive conclusions about long term oncological safety cannot be drawn.

**Table 5:** Oncological outcomes & margins

Outcome	Result
Follow-up Duration	Median 50 months (Range: 24–84)
Positive Margin Rate	0 / 23
Local Recurrence Rate	0 / 23
Distant Metastasis Rate	0 / 23
Re-operation Rate for Margins	0 / 23

Tumor response to NACT is heterogeneous, and chemoresistance remains a major obstacle to achieving pathological complete response, particularly in aggressive subtypes [25]. Understanding the molecular mechanisms of chemoresistance—including stem-like cell populations and alterations in apoptotic pathways—has become essential for optimizing treatment sequencing and surgical timing [26]. In our cohort, the high proportion of post neoadjuvant patients (73.9%) in our series reflects both institutional referral patterns for locally advanced disease and the specific clinical utility of PSI TM in this challenging context. Post NACT tissues may be more friable or fibrotic, yet our complication rates remained acceptable, suggesting PSI TM is well suited for this population.

Our Egyptian cohort may have different baseline breast morphology and cancer biology compared to Western populations. The applicability of PSI TM across diverse populations warrants investigation, particularly regarding differences in breast density, body habitus, and tumor characteristics. Multi-center international studies would better establish external validity. Furthermore, emerging evidence suggests that preoperative nutritional immunological indices may predict postoperative complications and survival in breast cancer patients [27]. Future studies should explore whether such indices can guide patient selection for PSI TM.

## Limitations and generalizability

Despite these strengths, limitations of the current study include the small sample size, single-center retrospective design, and variable follow up, which may overestimate safety and efficacy. Selection bias toward favorable anatomies (ptotic, larger breasts) limits generalizability, and the absence of direct comparators precludes definitive superiority claims over Grisotti. Aesthetic outcomes were assessed using a non-validated scale rather than validated instruments such as the BREAST Q or Harvard/Harris scale. Future studies employing these validated tools will provide more robust and comparable aesthetic data.

The application of PSI TM was deliberately limited to patients with cup size C or larger and Regnault grade II–III ptosis, as these anatomical features are essential for adequate flap mobility, volume redistribution, and tension free reconstruction. Consequently, this technique may not be suitable for patients with small breasts (cup A–B), minimal or no ptosis. We acknowledge that these strict selection criteria limit the generalizability of our findings and recognize that alternative oncoplastic approaches should be considered in such cases.

## Conclusion

In conclusion, PSI TM appears to be a feasible and safe oncoplastic technique for CLBC with NAC involvement, with theoretical vascular advantages and encouraging early outcomes. Prospective comparative studies are warranted to validate these findings.

## Data Availability

Data will be made available on a reasonable request.
